# Comparative analysis of intestinal microbiota composition between free-ranged captive yak populations in Nimu County

**DOI:** 10.3389/fcimb.2024.1420389

**Published:** 2024-06-25

**Authors:** Yanbin Zhu, Sijia Lu, Yangji Cidan, Hongzhuang Wang, Kun Li, Wangdui Basang

**Affiliations:** ^1^ Institute of Animal Husbandry and Veterinary Medicine, Tibet Academy of Agriculture and Animal Husbandry Sciences, Lhasa, China; ^2^ Linzhou Animal Husbandry and Veterinary Station, Lhasa, China; ^3^ College of Veterinary Medicine, Gansu Agricultural University, Lanzhou, China; ^4^ College of Veterinary Medicine, Nanjing Agricultural University, Nanjing, China; ^5^ MOE Joint International Research Laboratory of Animal Health and Food Safety, College of Veterinary Medicine, Nanjing Agricultural University, Nanjing, China

**Keywords:** captive yaks, free-range yaks, feeding style, gut microbiota, Nimu County, sequencing, Tibet autonomous region

## Abstract

The intestinal microbiota assumes a pivotal role in modulating host metabolism, immune responses, overall health, and additional physiological dimensions. The structural and functional characteristics of the intestinal microbiota may cause alterations within the host’s body to a certain extent. The composition of the gut microbiota is associated with environmental factors, dietary habits, and other pertinent conditions. The investigation into the gut microbiota of yaks remained relatively underexplored. An examination of yak gut microbiota holds promise in elucidating the complex relationship between microbial communities and the adaptive responses of the host to its environment. In this study, yak were selected from two distinct environmental conditions: those raised in sheds (NS, n=6) and grazed in Nimu County (NF, n=6). Fecal samples were collected from the yaks and subsequently processed for analysis through 16S rDNA and ITS sequencing methodologies. The results revealed that different feeding styles result in significant differences in the Alpha diversity of fungi in the gut of yaks, while the gut microbiota of captive yaks was relatively conserved. In addition, significant differences appeared in the abundance of microorganisms in different taxa, phylum *Verrucomicrobiota* was significantly enriched in group NF while *Firmicutes* was higher in group NS. At the genus level, *Akkermansia*, *Paenibacillus*, *Roseburia*, *Dorea*, *UCG_012*, *Anaerovorax* and *Marvinbryantia* were enriched in group NF while *Desemzia*, *Olsenella*, *Kocuria*, *Ornithinimicrobium* and *Parvibacter* were higher in group NS (P<0.05 or P<0.01). There was a significant difference in the function of gut microbiota between the two groups. The observed variations are likely influenced by differences in feeding methods and environmental conditions both inside and outside the pen. The findings of this investigation offer prospective insights into enhancing the yak breeding and expansion of the yak industry.

## Introduction

The intestinal microecology constitutes a dynamic ecosystem characterized by continuous fluctuations. Ruminant gastrointestinal tracts harbor vast populations of microorganisms, primarily comprising bacteria ((>98%), fungi (>0.1%), viruses, and protozoa (Larabi et al., 2020). In recent years, gut microbiota has been recognized as a signaling hub that integrates environmental inputs, genetic factors, and immune system signals to influence host metabolism, immunity, and infection responses (Thaiss et al., 2016). The composition of the gut microbiota is constantly shaped by multiple factors, such as dietary habits, seasons, lifestyle, antibiotics, or diseases (Rinninella et al., 2019). It is already established that the gut microbiota exerts a significant influence on animal digestive processes, with dietary composition standing out as a primary determinant in shaping the composition and function of the gut microbiota (Fan et al., 2023). The changes in gut microbiota also reflect the health status of the host, and the imbalance of gut microbiota has been associoated with various diseases (Wu et al., 2019).

Till now, research on the gut microbiota of ruminants were mainly focused on the type of animal, growth stages, and production performance. The composition and function of gut microbiota were explored through high-throughout sequencing analysis of their intestinal contents from different segments. Within diverse ruminant species, *Firmicutes* and *Bacteroidetes* emerge as predominant phyla, a pattern consistently observed across ruminants including sheep, yaks, and elk (Zeng et al., 2017; Xu et al., 2021; Lin et al., 2023). Furthermore, microbial communities within different segments of the intestine exhibit specificity, with the diversity and richness of microbial populations typically observed higher in the large intestine than that in the small intestine.

The yak, a distinctive cattle breed primarily inhabiting high-altitude regions (above 3000 meters), epitomizes a species evolved by natural selection, exhibiting remarkable adaptation to the cold, low-pressure, and hypoxic conditions prevalent in such environments. Its unique attributes render it an invaluable resource for the inhabitants of high-altitude areas (Li et al., 2020). It is generally believed that domesticating yaks originated in Xizang, China. Yaks are an important source of milk and meat products and play an important role in local economic development (Kong et al., 2024). In recent years, due to the promotion of sustainable and healthy development of ecological animal husbandry, the management of yak breeding has gradually shifted from year-round grazing to warm season grazing and cold season breeding in farms. However, many herders still adopt traditional grazing and breeding models. Exploring the impact of yak feeding patterns on the physical condition and economic value of yaks is crucial for the development of high-altitude areas. Previous studies have shown that cold and hypoxic environments may lead to changes in the gut microbiota composition (Ramos-Romero et al., 2020; Wang et al., 2020). Compared to the animals in plain areas, the high-altitude hypoxic environment in the Qinghai Tibet Plateau may shape distinct gut microbiomes in yaks (Wen et al., 2022). Therefore, this study explores the impact of different feeding models on the richness and diversity of yak gut microbiota, providing recommendations for improved feeding plans for yaks.

In this study, we utilized high-throughput sequencing (HTS) to investigate the effects of different feeding strategies on the gut microbiota structure and function of yaks in Nimu County. HTS is a molecular biology technique with high sequencing flux, fast detection speed, and low error rate, widely used in the field of microbiology research, and has been widely used in the exploration of gut microorganisms (Lee et al., 2020; Lu et al., 2023). The application of HTS in determining genomic sequences has been demonstrated for the first time through genome sequencing of *Acinetobacter baumannii*. Using this technology, we have explored the diversity, richness, structure, and functions of gut microbiota in yaks under different feeding conditions, to offer recommendations for the promotion of the healthy and sustainable development of the yak breeding industry.

## Materials and methods

### Sample collection

Fresh fecal samples were collected from 6 free-range yaks (group NF, n=6) and 6 captive yaks (group NS, n=6), belonging to herdsman and standardized yak farm, respectively, from Nimu County, Lhasa City, Xizang Autonomous Region, China (an average altitude above 3800 meters with an average annual temperature of 7°C). The fecal material was immediately collected after excretion using a sterile swab to avoid contamination and only the fresh feces were collected for subsequent testing. Fecal samples were snap-frozen using liquid nitrogen in sterile cryotubes and were stored at -80°C for subsequent testing.

### DNA extraction, PCR amplification, and library construction

Before DNA extraction, samples were vortexed for homogenization. OMEGA Soil DNA Kit (D5625–01) (Omega Bio-Tek, Norcross, GA, USA) was used to extract the genomic DNA (gDNA) from feces, quality and quantity of DNA was assessed and was stored at −20°C. According to the previous reports, the selected hypervariable regions of bacterial 16S rRNA genes (V3–V4) and fungal ITS genes were amplified by PCR using specific primers with Barcode and high-fidelity DNA polymerase (Lundberg et al., 2013; Wen et al., 2022; Lu et al., 2023). PCR products were subjected to 2% agarose gel electrophoresis, and DNA fragments were excised and purified by using Quant-iT Pico Green dsDNA Assay Kits.

Based on the preliminary quantitative results from electrophoresis, the recovered products of PCR amplification were detected and quantified using the Microplate reader (BioTek, FLx800) fluorescence quantitative system. The resulting proportions were combined according to the sequencing requirements of each sample. Library construction was performed using the TruSeq Nano DNA LT Library Prep Kit from Illumina. The quality of the prepared library was assessed using Agilent Bioanalyzer 2100 and Promega QuantiFluor. Once the library passed the quality control, it was subjected to sequencing.

### Bioinformatics and statistical analysis

The raw reads obtained in FASTQ format were pair-ended and preprocessed using cutadapt software to identify and remove adapters. Following trimming, the qualified paired-end reads from the previous step were subjected to quality filtering, noise reduction, splicing, chimerism removal, and other quality control analyses. These procedures were conducted according to the default parameters of QIIME 2 using DADA2 (Callahan et al., 2016; Bolyen et al., 2019), to obtain consensus sequences and ASV abundance tables. The consensus sequences were annotated and blasted against Silva database Version 138 using classify-sklearn with the default parameters. Based on taxonomic information, statistical analysis of community structure was conducted at various classification levels. Alpha and Beta diversity analysis was conducted to compare and analyze microbial community structure within and between communities, respectively (Lozupone et al., 2007; Mokany et al., 2011). LEFSe (Linear discriminant analysis effect size) and STAMP difference analysis were used to evaluate the significance level of species abundance difference and obtain the bacterial and fungal species information with significant differences between the two groups. PICRUSt2 (Phylogenetic Investigation of Communities by Recommendation of Unobserved States) was employed to predict the metabolic function of the flora based on marker gene (16S/ITS) sequence (Douglas et al., 2020).

The Wilcoxon test and ANOSIM were used to evaluate significant differences between different groups. *P* values < 0.05 were declared as statistically significant.

## Results

### Sequence analysis

**Figure 1:**
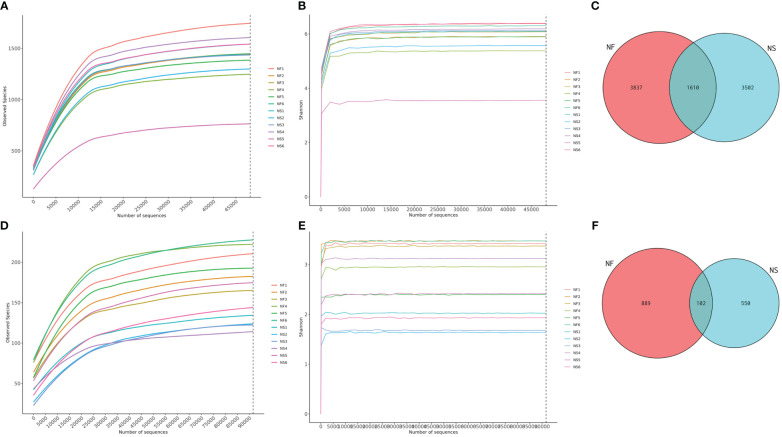
Sequencing data feasibility analysis and OTUs distribution. **(A)** Rarefaction curve of bacterial sequences, **(B)** Shannon curve of bacterial sequences, **(C)** Gut bacterial ASVs distribution in different groups, **(D)** Rarefaction curve of fungal sequences, **(E)** Shannon curve of fungal sequences, **(F)** Gut fungal ASVs distribution in different groups.

Twelve fecal samples were collected from free-range yaks (NF, n=6) and captive yaks (NS, n=6). Amplicon sequences of 16S and ITS were conducted to explore the differences in gut microbiota between yaks under captive feeding and grazing conditions. A total of over 1,711,000 raw data reads were obtained, with 848,297 for NF and 863,496 for NS in the bacterial analysis, and 853,474 for NF and 833,245 for NS in the fungal analysis (**Supplementary Tables 1**, **2**). After filtering, 1,640,000 sequences were retained for bacteria (NF=813,069, NS=827,157) and 1,623,000 for fungi (NF=810,811, NS=812,794). In this study, both sequencing results exhibited high-quality scores (Q20>95%, Q30>89%), suggesting high accuracy in base detection and meeting the requirements for further analysis. Additionally, rarefaction and Shannon curves showed a gradual flattening with the increasing number of sequencing data, indicating reasonable sequencing depth and a sufficiently large amount of data (**Figures 1A, B, D, E**). The sequences generated from the V3/4 and ITS regions were clustered into 8949 bacterial ASVs and 1541 fungal ASVs. Based on the clustering analysis results of ASVs, we analyzed common and unique ASVs between the two groups. Group NF yielded 3,837 bacterial ASVs, while group NS yielded 3,502 bacterial ASVs, with 1,610 ASVs shared between the two groups (**Figure 1C**). Additionally, 889 fungal ASVs were identified in group NF, and 550 fungal ASVs were identified in group NS, with 102 ASVs in common (**Figure 1F**).

### The effect of feeding styles on the diversity of yak Intestinal Flora

**Figure 2:**
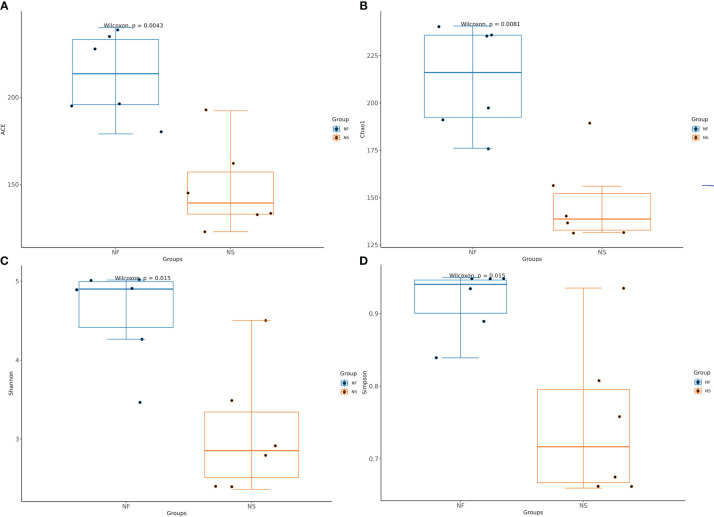
Comparative analysis of the Alpha diversity in gut fungi between house feeding and captive yaks. **(A)** ACE index, **(B)** Chao1 index, **(C)** Shannon index, **(D)** Simpson index.

**Figure 3:**
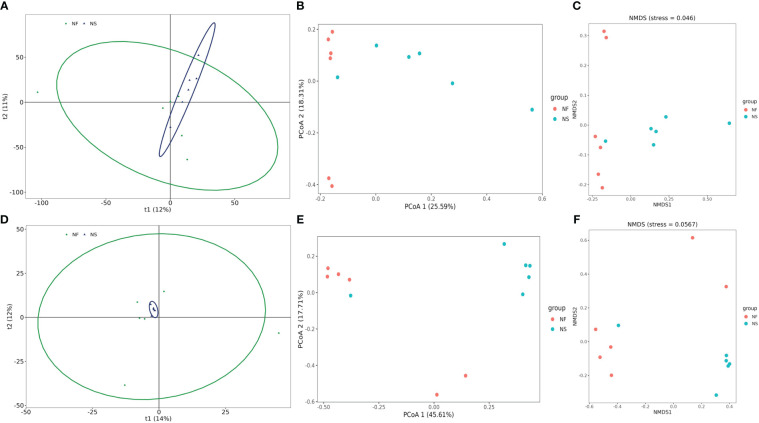
Comparative analysis of the gut bacterial and fungal beta diversities between house feeding and captive yaks. **(A, D)** gut bacterial and fungal PCA plots, **(B, E)** gut bacterial and fungal PCoA plots, **(C, F)** gut bacterial and fungal NMDS plots.

Alpha diversity and beta diversity of the yak gut microbial fraction were calculated to explore the effects of different feeding styles on the gut microbiota of yaks. The Chao1 estimator and ACE estimator were used to describe the richness of a community, while the Shannon index and Simpson index were used to show the diversity of species in the samples. There was no significant difference between the two groups in bacterial alpha diversity (**Table 1**). However, four fungal alpha diversity indices were significantly higher in group NF than in group NS (P<0.05) (**Table 2**), indicating that the richness and evenness of gut fungal community in grazing yaks were higher than those in captive yaks (**Figure 2**). Using the statistical algorithm Bray Curts for beta diversity analysis, the graph provides an intuitive observation of the distance between group samples. Samples with high similarity in community structure tend to cluster together, while samples with significant differences in community structure tend to be far apart. In the bacterial principal component analysis (PCA) plot (**Figure 3A**), dots representing the two groups are not distinctly dispersed, suggesting a less clear separation. In contrast, the fungal PCA plot (**Figure 3D**) exhibits a clear distinction between dots belonging to the two groups. PCoA analysis (**Figures 3B, E**) demonstrates that the distance between sample dots within the group is relatively close, while the distance between groups is relatively far in both bacterial and fungal analyses. This indicates noticeable independent clustering. The NMDS plot (**Figures 3C, F**) further supports these findings (ANOSIM, bacterial: p = 0.018, fungal: p=0.02).

**Table 1:** The indexes of alpha diversity of gut bacteria.

sample	observed_species	ACE	Chao1	Shannon	Simpson
NF1	1744	1810.216342	1813.068	9.2011	0.9944
NF2	1441	1489.527862	1496.598	8.5193	0.9826
NF3	1449	1501.954038	1511.16	8.768	0.9914
NF4	1248	1286.138338	1292.0323	7.762	0.9746
NF5	1386	1423.854067	1421.3465	8.5043	0.9876
NF6	1448	1489.130506	1483.9211	9.09	0.9955
NS1	1542	1594.533257	1586.7194	8.7926	0.9909
NS2	1301	1352.702004	1363.16	8.0396	0.981
NS3	1438	1478.330731	1479.4455	8.8396	0.9923
NS4	1607	1652.719655	1651.52	8.9251	0.991
NS5	765	802.3223353	809.0143	5.1313	0.8759
NS6	1543	1598.782962	1595.0238	9.222	0.9956

**Table 2:** The indexes of alpha diversity of gut fungi.

sample	observed_species	ACE	Chao1	Shannon	Simpson
NF1	211	234.6421	234.8824	4.9356	0.9344
NF2	183	194.9086	190.7727	5.0141	0.9497
NF3	166	179.0274	176.0588	4.8642	0.9462
NF4	223	228.6601	236.125	4.2639	0.8891
NF5	193	198.4924	197.4	3.4616	0.839
NF6	228	240.1577	240.65	5.0139	0.946
NS1	134	143.6319	140.5625	2.9206	0.6597
NS2	124	132.2439	131.5	2.3643	0.6645
NS3	123	135.0197	131.5	2.425	0.6749
NS4	114	122.9013	136.75	4.5044	0.9351
NS5	176	192.3004	189.1429	3.4846	0.8078
NS6	144	161.6346	156.0476	2.786	0.7583

These results suggest that different feeding styles have led to variations in the species composition structure of the yak gut microbiota community. The PCA, PCoA, and NMDS analyses collectively highlight the distinct microbial community patterns between yaks subjected to different feeding conditions.

### The effect of feeding regime on the community composition of intestinal bacteria

Based on the results of species annotation, choosing the top 10 taxa with the highest abundance ranking at levels of phylum and genus for each sample, a column accumulation chart of relative species abundance was generated to visualize the species with higher relative abundance and their proportion at different taxa.

**Figure 4:**
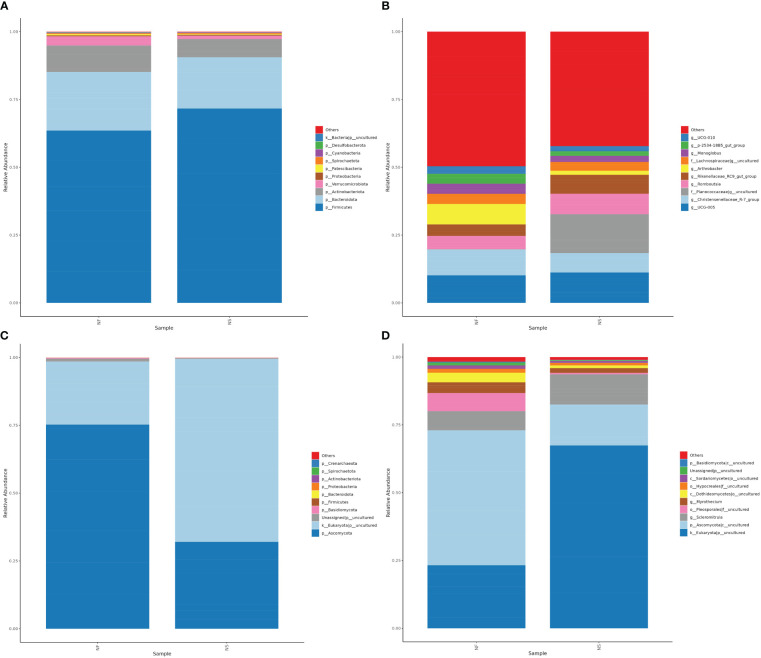
The relative abundance of bacterial **(A, B)** and fungal **(C, D)** taxa at the levels of Phylum and Genus.

At the phylum level, *Firmicutes* was the chief phyla in the two groups (NF: 63.52%, NS:71.65%) while it was more abundant in group NS. The phylum with the second highest abundance was *Bacteroidota* (NF: 21.62%, NS:18.91%), followed by *Actinobacteriota* (NF: 9.74%, NS:6.69%) (**Figure 4A**). The *Firmicutes*/*Bacteroidetes* ratio is often associated with body health (Magne et al., 2020), in this study, the *Firmicutes*/*Bacteroidetes* ratio was relatively higher in group NS. At the genus level, the primary genus in group NF was *UCG-005*, while the primary genera in group NS was an uncultured genus belonging to the family *Planococcaceae*, which was enricher in group NS (NF: 0.0837%%, NS:14.32%). In addition, the abundance of *Christensenellaceae_R-7_group* was higher in group NF while the abundance of *Romboutsia* was higher in group NS (**Figure 4B**).

### The effect of feeding regimes on the community composition of intestinal fungi

At the phylum level, *Ascomycota* has the top abundance in both groups (NF: 75.25%, NS:32.01%) in classified phylum, followed by *Basidiomycota* (NF: 0.37%, NS:0.18%) and *Firmicutes* (NF: 0.0233%, NS:0.0326%), together with uncultured phylum, they accounted for over 99.95% of the total number (**Figure 4C**). It is worth mentioning that a large number of unclassified fungi was detected in the intestines of domesticated yaks, which was significantly higher than that of grazing yaks. An unidentified family belonging to *Pleosporales* was more abundant in group NF (6.69%) than in group NS (0.53%). Genus *Scleromitrula* was much richer in group NS (11.13%) than group NF (7.05%) while *Myrothecium* was richer in group NF (3.94%) than in group NS (1.74%) (**Figure 4D**).

### Significant impact of different feeding regimes on intestinal microbiota

LEfSe analysis performs linear discriminant analysis (LDA) on samples according to different grouping conditions based on their taxonomic composition, identifying communities or species that have a significant impact on sample partitioning (Segata et al., 2011). In this study, LEfSe analysis was performed based on the abundance of species from two groups, species with LDA values > 2 were established biomarkers with statistical differences between groups.

**Figure 5:**
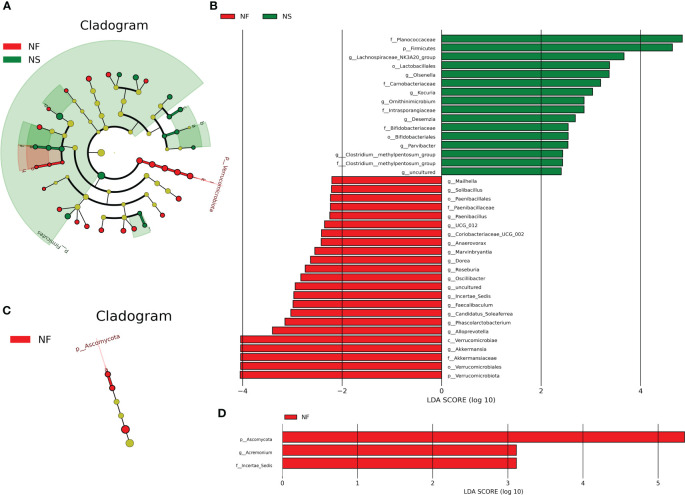
LEfSe analysis of intestinal bacterial **(A, B)** and fungal community **(C, D)** between house feeding and captive yaks at the levels of Phylum, Class, Order, Family, Genus, and Species.

A total of 39 biomarkers were detected in intestinal microbiota (**Figures 5A, B**). Phylum *Verrucomicrobiota* was significantly enriched in group NF, in this phylum, genus *Akkermansia* was significantly richer in NF, too. The abundance of Firmicutes was higher in group NS. In the class Bacilli belonging to Firmicutes, genus *Paenibacillus* was significantly richer in group NF while *Desemzia* was much more in group NS. In order Bacillales, genus *Solibacillus* was richer in group NF. In order *Oscillospirales* belonging to class *Clostridia*, *Oscillibacter*, *Candidatus_Soleaferrea* and *Incertae_Sedis* were enriched in group NF while *Clostridium:methylpentosum*_group was enriched in group NS. Besides, the abundance of genera *Roseburia*, *Dorea*, *UCG_012*, *Anaerovorax* and *Marvinbryantia* were higher in group NF. In addition, *Olsenella*, *Kocuria*, *Ornithinimicrobium* and *Parvibacter* were more abundant in group NS while *Alloprevotella*, *Coriobacteriaceae*_*UCG_002* and *Mailhella* were richer in group NF (P<0.05 or P<0.01). In intestinal fungi, the genus *Acremonium* belonging to *Iae_ncertSedis* in phylum *Ascomycota* was significantly richer in group NF (P<0.01) (**Figures 5C, D**).

### Metabolic alterations in gut microbiota to adapt to different feeding methods

**Figure 6:**
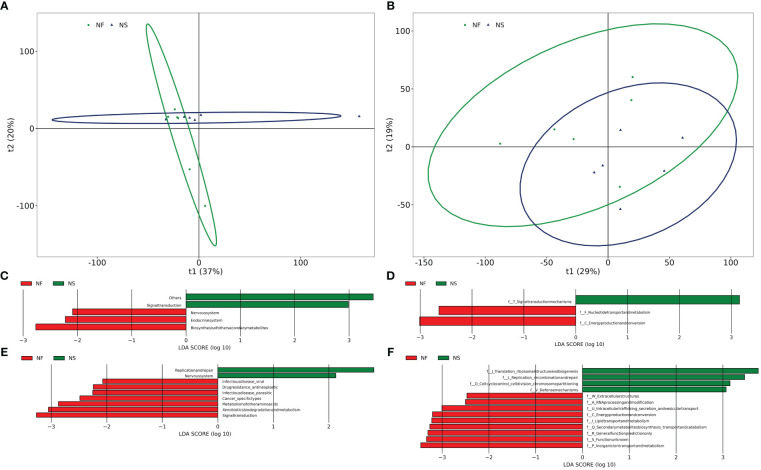
Comparative analysis of the function of gut bacterial **(A, C, E)** and fungal **(B, D, F)** between house feeding and captive yaks.

**Figure 7:**
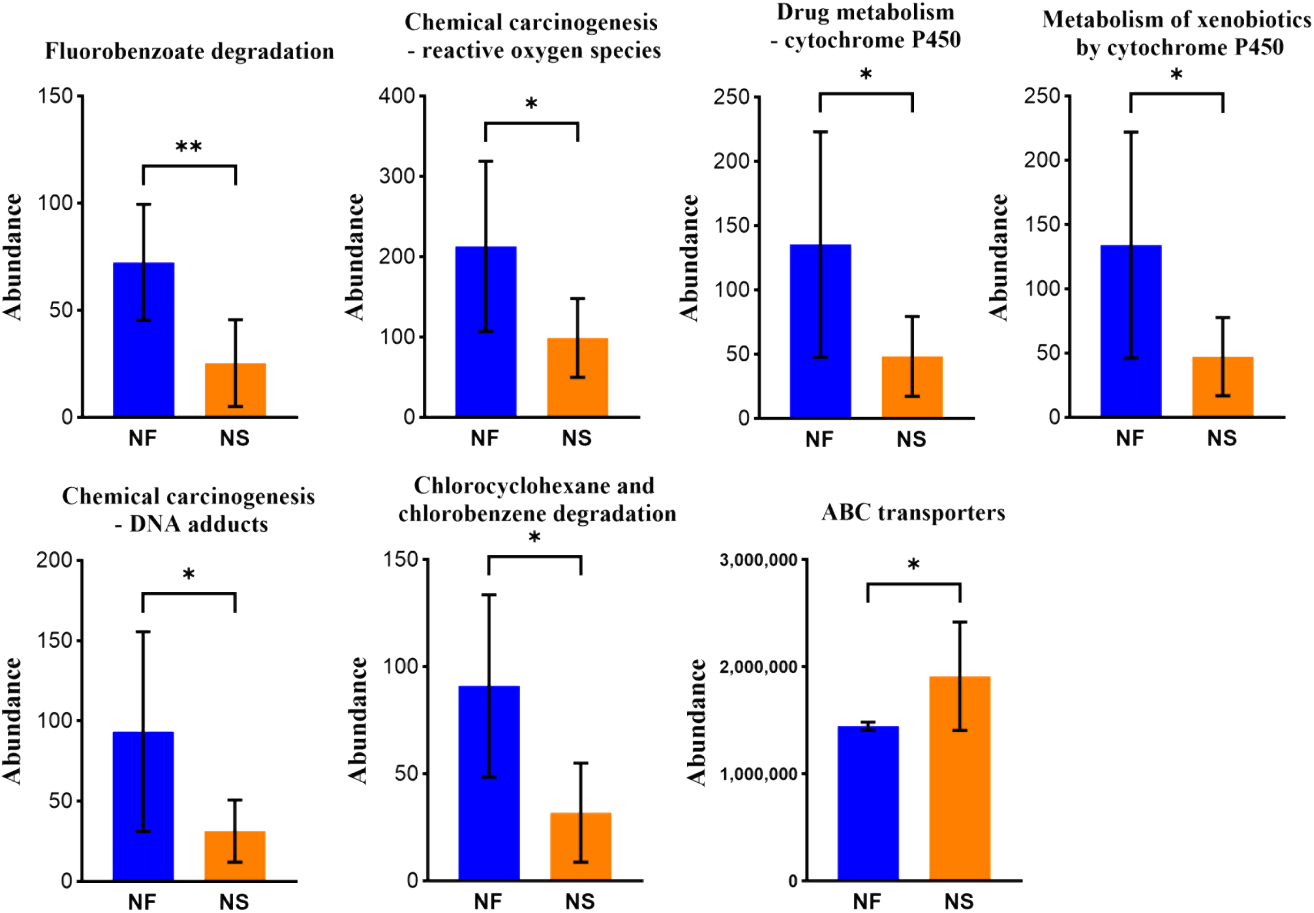
Iconic different pathways between two groups in metabolic pathways of KEGG l3. *P<0.05, **P<0.01.

PICRUSt was utilized to compare species composition information obtained from high-throughput sequencing data with information in the database, infer functional gene composition in samples, and analyze functional differences between various samples or groups (Ijoma et al., 2021). KEGG (Kyoto Encyclopedia of Genes and Genomes, primary database for studying metabolic pathways, integrating genomic information, chemical information, system information, and disease and health information (Kanehisa et al., 2017). COG (Clusters of Orthologous Groups) is a database for annotation of homologous proteins which can predict protein function well. PCA analysis revealed the similarity or difference in microbial community functions between the two groups on an overall level, results revealed that the bacterial functions were similar within the group, but more dispersed between groups while the dots of fungal functions were relatively scattered overall (**Figures 6A, B**). Results obtained from LDA combined with LEfSe analysis showed 13 significant differences in KEGG pathways between the two groups. Bacterial KEGG pathways including Biosynthesis of other secondary metabolites, Endocrine system, and nervous system were higher in group NF while Signal transduction was richer in group NS (P<0.05) (**Figure 6C**). Fungal KEGG pathways such as Signal transduction, Metabolism of other amino acids, and Cancer-specific types were distinctively richer in group NF while the “replication and repair,” nervous system was observed higher in group NS (P<0.05) (**Figure 6E**). The significant differences between the two groups in metabolic pathways of KEGG l3 are shown in **Figure 7**. 16 significant differences were recorded in COG function prediction between group NS and NF. Energy production and conversion, Nucleotide transport, and metabolism were found in higher tendency in group NF while signal transduction mechanisms were in group NS(P<0.05) in intestinal bacteria (**Figure 6D**). Energy production and conversion, Inorganic ion transport and metabolism, RNA processing and modification were detected richer in group NF while Replication_recombination and repair, Defense mechanisms were found higher in group NS (P<0.05 or P<0.01) in intestinal fungi (**Figure 6F**).

## Discussion

Yak (*Bos grunniens*) is one of the main domesticated cattle breeds in China, having dominant livestock species in the pastoral areas of the Qinghai Tibet Plateau since ancient times, renowned for its adaptability to high-altitude cold environments (>3000m above sea level). Domestic yaks are thought to have originated from Xizang, China, currently, two feeding modes viz. free range and artificial breeding are being practiced for domestic yaks. In recent years, many scholars have expressed their assertive view that factors such as diet and feeding environment shape the gut microbiota (Zmora et al., 2019). In yaks, there are also reports suggesting that different feeding modes/regimes have changed the structure and diversity of yak gut microbiota (Wen et al., 2022; Zhu et al., 2023).

In the current study, 16S and ITS rDNA sequencing analyses were employed to explore the differences in gut microbiota structure and function of yaks in Nimu County, Lhasa City under different feeding approaches. Fresh fecal samples of yaks belonging to the same age groups, yak farms, and free-ranged yaks were randomly collected for analysis. The results exhibited significant variations in the diversity, structure, and function of gut microbiota among two groups of yaks. Though no significant differences were observed from the results of bacterial alpha diversity of the two groups, the richness and evenness of gut fungal community in grazing yaks were found significantly higher than those in captive yaks. In addition, PCA plots showed that the aggregation phenomenon of points in the NS group can be observed, implying that the composition of gut microbiota in yaks fed in captivity was closely associated, especially with the fungal community. Captive yaks generally consume prepared feed (including alfalfa hay, oat hay, corn straw, and artificially prepared mixed feed), while the food for grazing yaks is undefined. Presumably, the regularity of diet and the stability of food ingredients are related to the relatively conservative gut microbiota of captive yaks, the complexity of environmental conditions may promote the increase of gut fungal richness in grazed yaks, the structural and functional changes of gut microbiota serve to enable yaks to adapt to the changing environment.

At the phylum level, Firmicutes and Bacteroidetes are widely recognized as the most abundant phyla in the gut microbiota of ruminants, including yaks (de Menezes et al., 2011; Nie et al., 2017; Zhang et al., 2020b). Results of the current study were consistent with this viewpoint of authors supported the already established data. They play a crucial role in the microbial ecology of ruminant intestines, for Firmicutes can effectively decompose lignin and cellulose, while Bacteroidetes can decompose nonfibrous feed material (Dowd et al., 2008; Shanks et al., 2011). Zhu et al. compared the intestinal microbiota of grazing and house-feeding yaks in Linzhou and found the grazing yaks had decreased level of Firmicutes(P<0.05) microbiota and relatively increased levels of Bacteroidetes, and a decreased ratio of Firmicutes/Bacteroidetes, which was consistent with results of the current study (Zhu et al., 2022). Artificially prepared food has relatively high protein, polysaccharide, and fat content, therefore house feeding yaks can steadily digest well-grounded, nutritious food. On the contrary, grazing yaks lack balanced feed, especially during the cold seasons around the year. Previous studies have shown a positive correlation between the relatively massive increase of Firmicutes and a highly nutritious diet. Bacteroides can promote decompose polysaccharides and proteins, improve the utilization rate of assimilated nutrients, and lead to improved digestion and absorption of nutrients (Zhang et al., 2020b). Therefore, it can be speculated that the enrichment of Firmicutes in the NF group was for better digestion of high-nutrient foods, while the elevation of Bacteroides in the NS group was for better utilization of the scarce food and adaptation to the environment with limited resources.

We further analyzed the differences in cultured bacteria between the two groups and found that 37 attributes showed significant changes. *Lachnospirace*ae sp. was related to the adaptation of high-grain diets (Chen et al., 2011), and higher abundance of Lachnospiraceae_NK3A20_group was found inbeef cattle fed with heated water in winter (He et al., 2023). Furthermore, Lachnospiraceae_NK3A20_group was significantly positively correlated with the rumen propionate and isovalerate (He et al., 2023). In addition, the same genus was associated to the digestion and absorption of proteins in the intestine, as well as the metabolism of glycerophospholipids, and the increased concentration of short-chain fatty acids (SCFAs) such as propionic acid (Liu et al., 2022; Ma et al., 2023). Compared to grazing yaks, house-feeding yaks existed in a warmer environment, with a higher protein and grain content in their diet. Thus increase of *Lachnospiraceae_NK3A20_group* was also a response of the gut microbiota to the feeding environment. Members of *Clostridium:methylpentosum_group* were also linked to the production of SCFAs (Himelbloom and Canale-Parola, 1989). Short chain fatty acids are the main products of intestinal microbes breaking down indigestible carbohydrates, and also generally considered as vital sources of energy to Propionate esters, which have the characteristics of reducing cholesterol, reducing fat storage, anti-cancer, and anti-inflammatory effects. Butyrate is crucial for maintaining the integrity of the intestinal wall and is also beneficial for the nervous system. Acetate helps to maintain intestinal pH to slightly acidic making it suitable for the colonization of beneficial microorganisms, and can also prevent opportunistic pathogenic bacteria from entering and adhering to intestinal epithelium (Lee et al., 2020; Moniri and Farah, 2021). Several genera belonging to *Lachnospiraceae* were found significantly increased in group NF, its members are the main source of short-chain fatty acids production, however, the different taxa of *Lachnospiraceae* were also related to different intestinal and extra-intestinal diseases. *Dorea* sp. can degrade polysaccharides, and oligosaccharides, directly related to glucose metabolism, and metabolizes to produce acetate (Palmnäs-Bédard et al., 2022). *Marvinbryantia* sp. and *Roseburia* sp. produce butyrate (Chen et al., 2021). In addition, genera belonging to other families such as *Faecalibaculum* and *Oscillibacter* increased in group NF, they are also producers of SCFAs. Among them, *Oscillibacter* was found in higher conc. Among the intestines of free-range yaks in Qinghai Province compared to captive feeding yaks (Wen et al., 2022), it was found in agreement with the results of the current studies. SCFAs produced by microorganisms can help resist the invasion of external pathogenic bacteria and alleviate intestinal diseases. Disconcertingly, several genera (*Paenibacillaceae*, *Verrucomicrobiota*, *Akkermansia*, *Ruminococcaceae*, *Marvinbryantia*, etc) which significantly increased in the NF group have been reported to be positively associated with metabolic diseases such as diabetes and obesity, including some SCFAs producers (Palmas et al., 2021; Rondanelli et al., 2021; Deledda et al., 2022). The abundance of Lactobacillus and *Olsenella* relatively decreased in group NF, while *Coriobacteriaceae*_ UCG_ 002 relative increased, similar tendencies were also found in the gut microbiota of animals with intestinal diseases such as colitis and inflammatory bowel disease (Zhang et al., 2020a; Zhang et al., 2023). It can be speculated that although the body has made adaptations, the complex environment in pastoral areas still has certain adverse effects on the health status of grazing yaks.

Although research on fungal microbiota is still in its early stages, fungi do have a significant impact on the homeostasis of gut microbiota, they coexist with bacterial microorganisms and greatly expand the gut microbiota that interacts with the intestinal immune system. Mainly, fungal microorganisms are responsible for decomposing lignocellulose in the intestine and achieving this function through the secretion of cell wall degrading enzymes and physical permeation (Wang et al., 2021). The significant enhancement of the phylum Ascomycota and genus Acremonium contributed to the unique gut fungal signature found in the NF group in this study (P<0.01). The enrichment of Ascomycota was previously found in yaks infected by *Cryptosporidium parvum* (Lu et al., 2023), grazed yaks are easily exposed to parasitic pathogens in the wild, and the significant increase of Ascomycota may suggest the potential presence of infection in yaks. However, metagenomic analysis was needed to further confirmation.

Differences in multiple metabolic pathways between two groups were found through microbial function prediction. Biosynthesis of other secondary metabolites and catabolism were detected richer in the grazing group. Microbial metabolites have effects on animal gut health and span cardiovascular and nervous systems as well (Thomas et al., 2022). The metabolic pathways related to the utilization of exogenous energy in grazing microorganisms are also prominent, in addition to significantly enriched nucleotide, lipid, and inorganic transport and metabolism. It is said that ruminants can utilize diet or endogenous nonprotein nitrogen (NPN) to furnish protein requirements. The aggregation of these functions by microorganisms also reflects their adaptation to food-deficient environments through efficient feed utilization. In addition, many disease-related metabolic pathways are also enhanced in the grazing group, such as cancer-specific types, infectious disease viral/parasitic, and drug resistance antineoplastic, we believe that living in pastoral areas increases the probability of yaks being exposed to pathogens and parasites. In group NS, the significant enriched defense mechanism and replication and repair were detected, implying that the microbial community of captive yaks has a certain degree of defense and self-repair ability, and a certain degree of conservatism and stability.

In current research, we found subtle significant differences in the gut microbiota of yaks under different feeding conditions, with different feed ingredients shaping the proportion and structure of microorganisms involved in nutrient utilization while dissimilar external environments also contribute to differences in microbial function and metabolism. The ecological environment and dietary structure are important driving factors for intestinal microbial succession, and the gut microbiota will change appropriately under various external stimuli to adapt to the habitat environment, further analysis of the connections between these variations and environmental factors is required.

## Conclusion

This study explored the composition and function of the intestinal microbiota in grazed yaks and house-feeding yaks and described the dynamic changes of the gut microbial community. The structure and diversity of the intestinal microflora of yaks were changed and such variation was more pronounced in fungal communities. Besides, we also found that variation in the abundance or function of certain microbe may be related to an adaptation to the external environment, including distinct alternations in some dominant phyla (Firmicutes and Ascomycota). Figuring out the effect of these factors on the intestinal microbial community can help us to understand the interaction mechanism between microbes and the host and the environment, provide a reference for better rearing of yaks, and a novel perspective for the development of prebiotics and microecological agents.

## Data availability statement

The datasets presented in this study can be found in online repositories. The names of the repository/repositories and accession number(s) can be found below: https://www.ncbi.nlm.nih.gov/, PRJNA1098018.

## Ethics statement

This study was guided and approved by the ethics committee of Nanjing Agricultural University (NJAU.No20220305025). The study was conducted in accordance with the local legislation and institutional requirements.

## Author contributions

YZ: Investigation, Writing – original draft. SL: Investigation, Writing – original draft. YC: Formal analysis, Methodology, Writing – original draft. HW: Data curation, Resources, Writing – original draft. KL: Conceptualization, Validation, Visualization, Writing – original draft, Writing – review & editing. WB: Conceptualization, Funding acquisition, Writing – original draft.

## Supplementary material

Supplementary Table 1 The data of 16S rRNA high-throughput sequencing.

Supplementary Table 2 The data of ITS genes high-throughput sequencing.
