# Supplementary material for: Comparative analysis of intestinal microbiota composition between free-ranged captive yak populations in Nimu County
Source: Front Cell Infect Microbiol. 2024 Jun 25;14:1420389. doi: 10.3389/fcimb.2024.1420389 (PMC11231391; doi:10.3389/fcimb.2024.1420389)
Supplement: Supplementary Table 1 — The data of 16S rRNA high-throughput sequencing. [file Table_1.docx]

Table S1 The data of 16S rRNA high-throughput sequencing

| sample-id | input | filtered | percentage of input passed filter | denoised | merged | percentage of input merged | non-chimeric | percentage of input non-chimeric | Total_ASVs | ASV_counts |
| --- | --- | --- | --- | --- | --- | --- | --- | --- | --- | --- |
| NF1 | 141115 | 135436 | 95.98 | 124230 | 75461 | 53.47 | 50281 | 35.63 | 8949 | 1744 |
| NF2 | 148888 | 142556 | 95.75 | 134529 | 93254 | 62.63 | 66304 | 44.53 | 8949 | 1441 |
| NF3 | 145659 | 139801 | 95.98 | 131795 | 88676 | 60.88 | 61145 | 41.98 | 8949 | 1449 |
| NF4 | 136537 | 131148 | 96.05 | 124893 | 89870 | 65.82 | 61715 | 45.2 | 8949 | 1248 |
| NF5 | 136395 | 130659 | 95.79 | 123397 | 83240 | 61.03 | 58448 | 42.85 | 8949 | 1386 |
| NF6 | 139703 | 133469 | 95.54 | 124162 | 78761 | 56.38 | 48182 | 34.49 | 8949 | 1448 |
| NS1 | 149030 | 143151 | 96.06 | 134076 | 89803 | 60.26 | 55257 | 37.08 | 8949 | 1542 |
| NS2 | 146120 | 140197 | 95.95 | 133321 | 98919 | 67.7 | 62715 | 42.92 | 8949 | 1301 |
| NS3 | 137613 | 131946 | 95.88 | 124545 | 84773 | 61.6 | 53028 | 38.53 | 8949 | 1438 |
| NS4 | 145005 | 138169 | 95.29 | 129699 | 90710 | 62.56 | 69568 | 47.98 | 8949 | 1607 |
| NS5 | 140316 | 134528 | 95.88 | 130207 | 109391 | 77.96 | 58071 | 41.39 | 8949 | 765 |
| NS6 | 145412 | 139166 | 95.7 | 130643 | 82971 | 57.06 | 52136 | 35.85 | 8949 | 1543 |

Table S2 The data of ITS genes high-throughput sequencing.

| sample-id | input | filtered | percentage of input passed filter | denoised | merged | percentage of input merged | non-chimeric | percentage of  input non-chimeric | Total_ASVs | ASV_counts |
| --- | --- | --- | --- | --- | --- | --- | --- | --- | --- | --- |
| NF1 | 142999 | 137461 | 96.13 | 136030 | 123041 | 86.04 | 116461 | 81.44 | 1541 | 211 |
| NF2 | 142260 | 138235 | 97.17 | 137186 | 126750 | 89.1 | 121163 | 85.17 | 1541 | 183 |
| NF3 | 142992 | 137559 | 96.2 | 136092 | 124179 | 86.84 | 122603 | 85.74 | 1541 | 166 |
| NF4 | 135046 | 122221 | 90.5 | 117568 | 95407 | 70.65 | 91600 | 67.83 | 1541 | 223 |
| NF5 | 140655 | 132334 | 94.08 | 128878 | 117701 | 83.68 | 103229 | 73.39 | 1541 | 193 |
| NF6 | 149622 | 143001 | 95.57 | 140418 | 132560 | 88.6 | 125101 | 83.61 | 1541 | 228 |
| NS1 | 140888 | 137443 | 97.55 | 136588 | 134340 | 95.35 | 115881 | 82.25 | 1541 | 134 |
| NS2 | 137879 | 134811 | 97.77 | 134314 | 131279 | 95.21 | 111654 | 80.98 | 1541 | 124 |
| NS3 | 144959 | 142038 | 97.98 | 141295 | 138416 | 95.49 | 118608 | 81.82 | 1541 | 123 |
| NS4 | 135608 | 131206 | 96.75 | 130391 | 125731 | 92.72 | 117244 | 86.46 | 1541 | 114 |
| NS5 | 135041 | 131841 | 97.63 | 131193 | 119582 | 88.55 | 96957 | 71.8 | 1541 | 176 |
| NS6 | 138870 | 135455 | 97.54 | 134894 | 130119 | 93.7 | 110200 | 79.35 | 1541 | 144 |
